# Efficacy and safety of olaparib, olaparib plus bevacizumab and niraparib maintenance treatment in Japanese patients with platinum-sensitive advanced ovarian cancer

**DOI:** 10.1093/jjco/hyad125

**Published:** 2023-09-14

**Authors:** Keiichiro Nakamura, Hirofumi Matsuoka, Masae Yorimitsu, Mariko Ogawa, Miho Kanemori, Kotaro Sueoka, Ayumi Kozai, Hiroko Nakamura, Tomoko Haruma, Yuko Shiroyama, Yuu Hayata, Hirokazu Sugii, Akiko Ueda, Shuichi Kurihara, Saiko Urayama, Miyuki Shimizu, Hisashi Masuyama

**Affiliations:** Department of Obstetrics and Gynecology, Okayama University Graduate School of Medicine, Dentistry and Pharmaceutical Sciences, Okayama, Japan; Department of Obstetrics and Gynecology, Okayama University Graduate School of Medicine, Dentistry and Pharmaceutical Sciences, Okayama, Japan; Department of Obstetrics and Gynecology, City Hiroshima Citizens Hospital, Hiroshima, Japan; Department of Obstetrics and Gynecology, National Organization Fukuyama Medical Center, Hiroshima, Japan; Department of Obstetrics and Gynecology, Fukuyama City Hospital, Hiroshima, Japan; Department of Obstetrics and Gynecology, Yamaguchi University Graduate School of Medicine, Yamaguchi, Japan; Department of Perinatology and Gynecology, Kagawa University Graduate School of Medicine, Kagawa, Japan; Department of Obstetrics and Gynecology, National Hospital Organization KURE Medical Center and Chugoku Cancer Center, Hiroshima, Japan; Department of Obstetrics and Gynecology, Saiseikai General Hospital, Okayama, Japan; Department of Obstetrics and Gynecology, Prefectural Hospital, Hiroshima, Japan; Department of Obstetrics and Gynecology, Kagawa Prefectural Central Hospital, Kagawa, Japan; Department of Obstetrics and Gynecology, National Hospital Organization Iwakuni Clinical Center, Yamaguchi, Japan; Department of Obstetrics and Gynecology, Onomichi General Hospital, Hiroshima, Japan; Department of Obstetrics and Gynecology, Japanese Red Cross Matsuyama Hospital, Ehime, Japan; Department of Obstetrics and Gynecology, Higashi Hiroshima Medical Center, Hiroshima, Japan; Department of Obstetrics and Gynecology, Kagawa Rosai Hospital, Kagawa, Japan; Department of Obstetrics and Gynecology, Okayama University Graduate School of Medicine, Dentistry and Pharmaceutical Sciences, Okayama, Japan

**Keywords:** olaparib, olaparib plus bevacizumab, niraparib, renal function

## Abstract

**Objective:**

To investigate whether maintenance treatment could be safely and effectively performed with olaparib, olaparib plus bevacizumab and niraparib in platinum-sensitive advanced ovarian cancer at multiple institutions in Japan.

**Methods:**

We investigated progression-free survival and adverse events in 117 patients with platinum-sensitive advanced ovarian cancer treated with maintenance therapy.

**Results:**

The median progression-free survival of 117 patients was 20.1 months. Patients with germline BRCA pathogenic variants had a significantly better prognosis than the other groups (*P* < 0.001). Furthermore, in the multivariate analysis, stage IV (*P* = 0.016) and germline BRCA wild-type (*P* ≤ 0.001) were significantly associated with worse progression-free survival in patients with advanced ovarian cancer. Regarding adverse events, all three types of maintenance treatment were significantly worse than chemotherapy given before maintenance treatment with respect to renal function (olaparib, *P* = 0.037; olaparib plus bevacizumab, *P* < 0.001; and niraparib, *P* = 0.016).

**Conclusion:**

Maintenance treatment was performed effectively and safely. Renal function deterioration is likely to occur during maintenance treatment, and careful administration is important in platinum-sensitive advanced ovarian cancer.

## Teaser

Maintenance treatment in patients with platinum-sensitive advanced ovarian cancer is likely to cause deterioration of renal function, and careful administration is essential.

## Introduction

Epithelial ovarian cancer (OC) is the sixth most common cancer among women worldwide and the leading cause of death due to gynecological malignancies (1). Over decades, systemic chemotherapy regimens for OC have evolved, and molecular targeted drugs have been used in concomitant chemotherapy and maintenance therapy. Recently, poly (ADP-ribose) polymerase inhibitors (PARPi) have been approved and used as maintenance treatment drugs for patients with advanced OC (stages III–IV) who respond to platinum-based chemotherapy in the initial treatment stages. These agents exploit BRCA variants and DNA damage-response deficiencies. Inhibition of PARP leads to propagation of single-strand DNA breaks and the accumulation of double-strand breaks, which require repair by homologous recombination repair mechanisms. Germline BRCA (gBRCA) testing is used as a companion diagnostic tool for PARPi therapy in treating advanced OC in Japan. BRCA1/2-mutated tumors and those with other forms of homologous recombination deficiency (HRD) are particularly susceptible to PARP inhibition and have seen the greatest benefits in response rates and progression-free survival (PFS) in clinical trials. PARPi use as maintenance therapy in the front-line setting is now considered the standard of care in patients with BRCA1/2 mutations based on the SOLO-1/GOG-3004/ENGOT study (2). PARP inhibitors are also recommended as per the American Society of Clinical Oncology guidelines in all patients with OC as front-line maintenance therapy based on the PRIMA/ENGOT-OV26/GOG-3012 trial (3). The combination of olaparib PARPi and bevacizumab anti-angiogenesis inhibitor is also approved as maintenance therapy following front-line chemotherapy treatment in patients with HRD tumors and is an option for patients who have initiated bevacizumab with their chemotherapy treatment based on the PAOLA-1/ENGOT-ov25 trial (4). Olaparib was approved in June 2019 as maintenance therapy after initial treatment of BRCA-positive OC, and was approved in December 2019 as maintenance therapy with niraparib with or without BRCA. In December 2020, HRD measurement was added, and excised tissue is now submitted for the selection of maintenance treatment drugs. Currently, HRD or BRCA status is measured and treatment is determined according to gynecological guidelines. However, there have been no reports of the actual use of the three maintenance treatments (olaparib, olaparib plus bevacizumab and niraparib) in clinical practice in Japan. We investigated the current clinical situation to ensure effective and safe PARPi maintenance treatment in Japan.

## Patients and methods

In this multicenter retrospective study of 117 patients with platinum-sensitive advanced OC, all patients started PARPi (olaparib, olaparib plus bevacizumab or niraparib) maintenance treatment from January 2019 to March 2023. This study was approved by the ethics committee of Okayama University (2203–006). The subjects were Japanese patients aged 20 years old or older who met the following inclusion criteria: (i) patients with a complete response (CR) or partial response (PR) after successful platinum-based chemotherapy; and (ii) patients with adequate function of the bone marrow and major organs.

Patients’ germline status of *BRCA* was determined by testing baseline blood samples in a central laboratory in Japan using the Myriad BRACAnalysis CDx® test (Salt Lake City, UT, USA). The gBRCA results were classified as pathogenic variants (deleterious gBRCA1 and/or gBRCA2 pathogenic variants; genetic variants suspected to be deleterious), VUS (variant of unknown significance) or wild-type (gBRCA wild-type; genetic variant of uncertain significance; genetic variant, favor polymorphism; no pathogenic variant/deleterious pathogenic variant detected). Myriad myChoice CDx defines an HRD-positive status as deleterious or suspected deleterious mutations in *BRCA1* and *BRCA2* genes and/or positive genomic instability, which is calculated by the genomic instability score (GIS). The GIS is an algorithmic measurement of loss of heterozygosity, telomeric allelic imbalance and large-scale state transitions. A GIS of ≥42 is considered positive for HRD status, whereas a GIS of <42 (biomarker-negative) suggests that the homologous recombination pathway is not defective.

Patients were excluded on the basis of the following criteria: (a) patients with a history of PARPi treatment; (b) patients with a history of hypersensitivity to the ingredients of PARPi; (c) patients who had received abdominal radiotherapy; (d) patients with severe bone marrow suppression complications; (e) patients with ovarian borderline malignant tumors; and (f) patients with history of other clinically active malignancies within 5 years of enrollment.

### Study design

This was a retrospective observational study that was conducted in the setting of routine clinical practice without any special interventions. The dosage and administration, premedication, dose reduction/interruption/discontinuation and examinations (including imaging studies) were all performed according to the SOLO1, PAOLA-1/ENGOT-ov25 and PRIMA/ENGOT-OV26 trials in principle (2–4). Patients received olaparib and niraparib orally after they had completely recovered from their last platinum-based chemotherapy. Olaparib and niraparib were interrupted if neutrophil counts were <1000/mm^3^, hemoglobin was <8.0 g/dl, platelet counts were < 50 000/mm^3^ or estimated glomerular filtration rate (eGFR) was <30 ml/min/1.73m^2^. Treatment was restarted if the neutrophil count increased to ≥1500/mm^3^, hemoglobin was ≥9.0 g/dl, platelet counts were ≥ 75 000/mm^3^ or eGFR was ≥30 ml/min/1.73 m^2^.

The primary endpoints were related to efficacy, including the PFS time and the response rate in the primary analysis cohort. The second endpoint was the incidence of maintenance therapy-induced hematological toxicities (anemia, neutropenia, thrombocytopenia) and renal function. The incidences of grade ≥ 3 hematologic toxicity and grade ≥ 2 renal function (eGFR) adverse events (AEs) was reviewed in detail.

Exploratory endpoints were the incidence rates of important PARPi-specific AEs in the exploratory analysis cohort. AEs were evaluated according to the Common Terminology Criteria for Adverse Events Version 4.0 (5), and the frequency of the most severe grade of each event in each patient during all treatment cycles was calculated. Tumor response and disease progression were evaluated by computed tomography or magnetic resonance imaging according to the new Response Evaluation Criteria in Solid Tumours (revised RECIST guideline, version 1.1) (6) by each investigator. Recurrence was determined using imaging methods (according to RECIST, version 1.1) at regular intervals or upon the onset of symptoms. PFS was defined as the time from randomization after completion of platinum-based chemotherapy to objective disease progression on imaging studies. The response rate was calculated as the percentage of patients in the analysis cohort with a measurable lesion in whom the best overall response according to RECIST was a CR or a PR.

### Statistical analysis

Statistical analyses were performed using the Mann–Whitney U test for comparisons with controls, and one-factor ANOVA followed by Fisher’s protected least significance difference test for all pairwise comparisons. PFS rates were calculated using the Kaplan–Meier method, and differences between the survival curves were examined using the log-rank test. The analyses were performed using StatView software (version 25.0; Abacus Concepts, Berkeley, CA, USA). Differences were considered statistically significant at *P* < 0.05.

## Results

We investigated the distribution of olaparib-, olaparib plus bevacizumab- and niraparib-treated patients for each of the following clinical characteristics: age, body mass index (BMI), stage, histology, primary debulking surgery (PDS), interval debulking surgery (IDS), gBRCA status, HRD status and response to last platinum therapy. Age, BMI, stage, histology, PDS, IDS, gBRCA status, HRD status and response to last platinum therapy for the three groups of olaparib, olaparib plus bevacizumab and niraparib are detailed in Table 1.

**Table 1:** Patient and tumor characteristics

Baseline characteristics				
		Olaparib (*N* = 30)	Olaparib plus bevacizumab (*N* = 27)	Niraparib (*N* = 60)
**Age**				
	≦49	7 (23.3%)	4 (14.8%)	7 (11.7%)
	50–59	6 (20.0%)	7 (25.9%)	13 (21.7%)
	60–69	9 (30.0%)	8 (29.6%)	17 (28.3%)
	70–79	7 (23.3%)	8 (29.6%)	19 (31.7%)
	≧80	1 (3.3%)	0 (0.0%)	4 (6.7%)
**BMI**				
	<18.5	4 (13.3%)	1 (3.7%)	7 (11.7%)
	18.5–24.9	22 (73.3%)	21 (77.8%)	40 (66.7%)
	25.0–29.9	3 (10.0%)	4 (14.8%)	10 (16.7%)
	30.0–34.9	1 (3.3%)	1 (3.7%)	3 (5.0%)
	≧35.0	0 (0.0%)	0 (0.0%)	0 (0.0%)
**Stage**				
	III	18 (60.0%)	19 (70.4%)	29 (48.3%)
	IV	12 (40.0%)	8 (29.6%)	31 (51.7%)
**Histology**				
	High-grade serous carcinoma	30 (100.0%)	24 (88.9%)	42 (70.0%)
	Endometrioid carcinoma	0 (0.0%)	1 (3.7%)	5 (8.3%)
	Clear cell carcinoma	0 (0.0%)	0 (0.0%)	3 (5.0%)
	Other types carcinoma	0 (0.0%)	2 (7.4%)	3 (5.0%)
	Unclassified carcinoma	0 (0.0%)	0 (0.0%)	7 (11.7%)
				
**Primary debulking surgery**				
	Present	8 (26.7%)	9 (33.3%)	9 (15.0%)
	Absent	22 (73.3%)	18 (66.7%)	51 (85.0%)
**Interval debulking surgery**				
	R0	12 (40.0%)	15 (55.5%)	24 (40.0%)
	R1	10 (33.3%)	11 (40.7%)	16 (26.7%)
	R2	6 (20.0%)	1 (3.7%)	9 (15.0%)
	Unknown	2 (6.7%)	0 (0.0%)	11 (18.3%)
**Germline BRCA1/2 status**				
	Pathogenic variant	30 (100.0%)	7 (25.9%)	0 (0.0%)
	VUS	0 (0.0%)	0 (0.0%)	1 (1.7%)
	Wild type	0 (0.0%)	8 (29.6%)	24 (40.0%)
	Not analyzed	0 (0.0%)	12 (44.4%)	35 (58.3%)
**Tumor HRD status**				
	HRD&tBRCA-	0 (0.0%)	17 (63.0%)	2 (3.3%)
	HRD&tBRCA+	4 (13.3%)	10 (37.0%)	0 (0.0%)
	HRP	0 (0.0%)	0 (0.0%)	14 (23.3%)
	Not analyzed	26 (86.7%)	0 (0.0%)	44 (73.3%)
**Response to the last platinum therapy**				
	Complete response	20 (66.7%)	21 (77.8%)	37 (61.7%)
	Partial response	10 (33.3%)	6 (22.2%)	23 (38.3%)

BMI, body mass index; VUS, variant of unknown significance; HRD: homologous recombination deficient.

**Figure 1:**
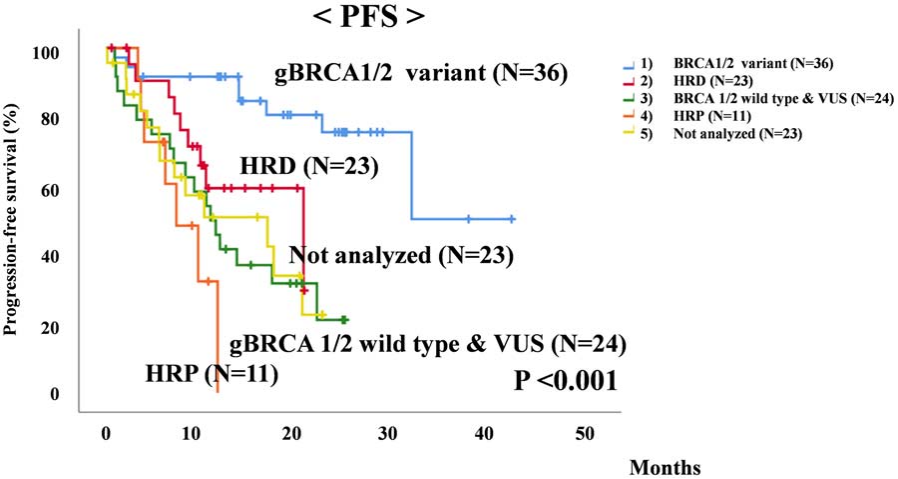
Comparison of progression-free survival (PFS) in patients with germline BRCA (gBRCA) pathogenic variants, homologous recombination deficiency (HRD), gBRCA wild-type, variant of unknown significance (VUS), homologous recombination proficiency (HRP) and not analyzed patients with maintenance treatment for platinum-sensitive advanced ovarian cancer.

In this study, we investigated PFS by BRCA status and HRD status, and PFS by each maintenance treatment. HRD and gBRCA pathogenic variants in gBRCA searches are classified as gBRCA pathogenic variants. Patients who were HRD-positive and tBRCA-negative, tBRCA-positive with gBRCA-wild-type or with no gBRCA testing were classified as HRD-positive. A total of 30 patients (30 with gBRCA pathogenic variants) received olaparib. Olaparib plus bevacizumab was administered to a total of 27 patients: 6 patients with gBRCA pathogenic variants, 4 who were HRD-positive and had tBRCA with gBRCA wild-type and 17 who were HRD-positive and tBRCA-negative. In total, 60 patients received niraparib: 11 homologous recombination proficiency (HRP) patients, 2 HRD-positive and tBRCA-negative patients, 23 not analyzed patients and 24 gBRCA-wild-type and VUS patients. The median PFS of 117 patients who underwent maintenance treatment for platinum-sensitive advanced OC was 20.1 months (95% confidence interval [CI] 15.0–25.2). For PFS with respect to BRCA and HRD status, gBRCA pathogenic variant patients did not reach the median. The median PFS of patients with HRD status was 20.2 months (95% CI 5.9–34.6), while that of patients with gBRCA wild-type and VUS was 10.7 months (95% CI 7.5–13.9), 7.2 months (95% CI 3.3–11.1) for patients with HRP and 16.5 months (95% CI 3.0–30.1) for patients with unknown status. Therefore, patients with gBRCA pathogenic variants had a significantly better prognosis than the other groups of HRD, gBRCA wild-type and VUS, HRP and not analyzed patients (*P* < 0.001; Fig. 1A). However, in the examination of PFS in maintenance treatment, olaparib and olaparib plus bevacizumab did not reach the median value, as with patients with gBRCA pathogenic variants. The median PFS of patients with niraparib was 10.3 months (95% CI 7.0–13.5).

Correlations between clinical factors and PFS were assessed by univariate and multivariate analyses. In the univariate analysis, stage (*P* = 0.012) and gBRCA wild-type (including VUS) (*P* < 0.001) were significantly associated with worse PFS. In the multivariate analysis, stage (*P* = 0.016) and gBRCA wild-type (*P* < 0.001) were significantly associated with worse PFS in patients with advanced OC. On the basis of these results, we concluded that maintenance therapy is greatly affected by gBRCA status (Table 2).

**Table 2:** Prognostic factors for PFS selected by Cox’s univariate and multivariate analysis

	Exp(B)	95% CI	Cox’s test	Exp(B)	95% CI	Cox’s test
			*P* value			*P* value
Age (≧ 70 years)	1.393	0.804–2.413	0.237			
BMI (<18.5)	1.62	0.731–3.591	0.235			
Stage (≧stage IV)	1.994	1.166–3.411	0.012^*^	1.935	1.128–3.317	0.016^*^
Histology (HGSC)	0.691	0.369–1.293	0.248			
HRP	2.006	0.940–4.280	0.072			
gBRCA wild type (include VUS)	4.654	2.152–10.065	<0.001^*^	4.583	2.116–9.923	<0.001^*^
PDS absent	1.352	0.680–2.689	0.39			
Interval debulking surgery (≧R1)	1.536	0.878–2.688	0.133			
Response to the last platinum therapy (PR)	1.552	0.901–2.673	0.114			

BMI: body mass index, HGSC: high-grade serous carcinoma, HRP: homologous recombination proficient, PR: partial response, ^*^P < 0.05

We next investigated the distribution of each of the AEs of maintenance treatment, including first occurrence of AEs, AEs resulting in drug interruption or discontinuation, number of drug interruptions, dosage reduction of maintenance treatment and duration of maintenance treatment. First occurrence of AEs occurred within ~3 months in all three groups. For AEs resulting in drug interruption or discontinuation, olaparib- and olaparib plus bevacizumab-treated patients were significantly more anemic than those treated with niraparib (*P* = 0.044 and *P* = 0.001, respectively), and niraparib-treated patients were significantly more thrombocytopenic than olaparib-treated patients (*P* = 0.005). In total, 46.7–51.9% of patients treated with olaparib, olaparib plus bevacizumab or niraparib had their treatment doses reduced. In particular, niraparib was discontinued due to thrombocytopenia within 1 month, and cases of withdrawal were observed (Table 3).

**Table 3:** Adverse events by maintenance treatment

Baseline characteristics			Olaparib (*N* = 30)	Olaparib plus bevacizumab (*N* = 27)	Niraparib (*N* = 60)
**First occurrence of adverse events time**					
	< 1 month		12 (40.0%)	7 (25.9%)	23 (38.3%)
	1–3 months		5 (16.7%)	10 (37.0%)	4 (6.7%)
	4–6 months		2 (6.7%)	0 (0.0%)	0 (0.0%)
	> 6 months		1 (3.3%)	2 (7.4%)	0 (0.0%)
**Adverse events of drug interruption or discontinuation (Include duplicates)**					
	Anemia		10 (33.4%)	13 (48.1%)	9 (15.0%)
	Neutropenia		5 (16.7%)	3 (11.1%)	4 (6.7%)
	Thrombocytopenia		1 (3.3%)	4 (14.8%)	17 (28.3%)
	Nausea		1 (3.3%)	2 (7.4%)	2 (3.3%)
	Fatigue		3 (10.0%)	1 (3.7%)	2 (3.3%)
	Other Adverse events		3 (10.0%)	1 (3.7%)	6 (10.0%)
**Number of drug interruptions**					
	1		8 (26.7%)	6 (22.2%)	23 (38.3%)
	2		6 (20.0%)	5 (18.5%)	8 (13.3%)
	3		3 (10.0%)	4 (14.8%)	0 (0.0%)
	≧4		3 (10.0%)	4 (14.8%)	1 (1.7%)
**Dosage reduction of maintenance treatment**					
	Absent		16 (53.3%)	13 (48.1%)	32 (53.3%)
	Present		14 (46.7%)	14 (51.9%)	28 (46.7%)

In this study, we decided to examine hematological toxicities and kidney function during maintenance treatment and during chemotherapy given before maintenance treatment in platinum-sensitive advanced OC. Using grade 3 as the discontinuation criterion for hematological toxicities in maintenance treatment, subjects were divided into two groups: less than grade 2 and grade 3 or higher. In the olaparib plus bevacizumab group, anemia was significantly worse in those treated with maintenance treatment than in those who received chemotherapy (*P* = 0.013). Conversely, neutropenia following all three types of chemotherapy was significantly worse than that following maintenance treatments (*P* = 0.006, *P* = 0.020 and *P* < 0.001, respectively).

The use of PARP inhibitors may lead to decreased renal function. Olaparib has been shown to inhibit multidrug and toxin extruders 1 and 2 and organic cation transporters 1 and 2, and niraparib is known to function without interacting with the proximal tubular transporter responsible for PARPi secretion. (7). Gupt et al. compared the renal function of olaparib and niraparib, and reported that although both showed decreased renal function, there was no difference between the two drugs (8). We investigated the correlation in kidney function between maintenance treatment and chemotherapy given before maintenance treatment. The subjects were divided into two groups: less than grade 1 and grade 2 or higher. All three types of maintenance therapy were associated with significantly worse renal function than chemotherapy (*P* = 0.037, *P* < 0.001 and *P* = 0.016, respectively) (Table 4).

**Table 4:** Relationship between adverse events of maintenance treatment and chemotherapy given before maintenance treatment

		Chemotherapy	Maintenance treatment		Chemotherapy	Maintenance treatment		Chemotherapy	Maintenance treatment	
	Grade	Olaparib (*N* = 30)	Olaparib (*N* = 30)	*P* value	Olaparib plus bevacizumab (*N* = 27)	Olaparib plus bevacizumab (*N* = 27)	*P* value	Niraparib (*N* = 60)	Niraparib (*N* = 60)	*P* value
**Anemia**				0.39			0.013^*^			0.793
	1	12 (40.0%)	9 (30.0%)		8 (29.6%)	10 (37.1%)		17 (28.3%)	30 (50.0%)	
	2	11 (36.7%)	11 (36.7%)		16 (59.3%)	6 (22.2%)		35 (58.3%)	21 (35.0%)	
	3	7 (23.3%)	10 (33.3%)		3 (11.1%)	11 (40.7%)		8 (13.3%)	9 (15.0%)	
**Neutropenia**				0.006^*^			0.020^*^			<0.001^*^
	0	2 (6.7%)	8 (26.7%)		2 (7.4%)	6 (22.2%)		7 (11.7%)	18 (30.0%)	
	1	6 (20.0%)	5 (16.7%)		3 (11.1%)	7 (25.9%)		11 (18.3%)	21 (35.0%)	
	2	7 (23.3%)	12 (40.0%)		9 (33.3%)	9 (33.3%)		14 (23.3%)	15 (25.0%)	
	3	10 (33.3%)	5 (16.7%)		3 (11.1%)	5 (18.5%)		11 (18.3%)	6 (10.0%)	
	4	5 (16.7%)	0 (0.0%)		10 (37.1%)	0 (0.0%)		17 (28.3%)	0 (0.0%)	
**Thrombocytopenia**				0.15			0.552			0.17
	1	24 (80.0%)	26 (86.7%)		24 (88.9%)	24 (88.9%)		54 (90.0%)	53 (88.3%)	
	2	4 (13.3%)	4 (13.3%)		2 (7.4%)	1 (3.7%)		5 (8.3%)	3 (5.0%)	
	3	2 (6.7%)	0 (0.0%)		1 (3.7%)	2 (7.4%)		1 (1.7%)	1 (1.7%)	
	4	0 (0.0%)	0 (0.0%)		0 (0.0%)	0 (0.0%)		0 (0.0%)	3 (5.0%)	
**eGFR**				0.037^*^			<0.001^*^			0.016^*^
	0	12 (40.0%)	5 (16.7%)		14 (51.9%)	2 (7.4%)		32 (53.3%)	13 (21.7%)	
	1	9 (30.0%)	8 (26.7%)		7 (25.9%)	5 (18.5%)		9 (15.0%)	15 (25.0%)	
	2	8 (26.7%)	16 (53.3%)		6 (22.2%)	19 (70.4%)		19 (31.7%)	29 (48.3%)	
	3	0 (0.0%)	0 (0.0%)		0 (0.0%)	1 (3.7%)		0 (0.0%)	3 (5.0%)	
	4	1 (3.3%)	1 (3.3%)		0 (0.0%)	0 (0.0%)		0 (0.0%)	0 (0.0%)	

^*^ *P* < 0.05

## Discussion

The SOLO-1/GOG-3004/ENGOT, PAOLA-1/ENGOT-ov25 and PRIMA/ENGOT-OV26/GOG-3012 trials demonstrated the efficacy of maintenance treatment in platinum-sensitive advanced OC (2–4). In the SOLO1 trial, PFS was significantly longer in olaparib-treated patients (not evaluable; hazard ratio [HR] 0.30, 95% CI 0.23–0.41) than in the controls (13.8 months) (2). In the PAOLA-1 trial, the median follow-up for the PFS of all variables, BRCA pathogenic variants, HRD positivity, HRD-positive non-BRCA pathogenic variants and HRD negativity, was significantly longer for olaparib plus bevacizumab (22.1 months: HR 0.59, 95% CI 0.49–0.72; 37.2 months: HR 0.31, 95% CI 0.20–0.47; 37.1 months: HR 0.33, 95% CI 0.25–0.45; 28.1 months: HR 0.43, 95% CI 0.28–0.66; and 16.6 months: HR 1.00, 95% CI 0.75–1.35, respectively) than that of the control (16.6, 21.7, 17.7, 16.6 and 16.2 months, respectively) (4). In the PRIMA trial, the median follow-up for PFS of all variables, BRCA pathogenic variants, HRD positivity, HRD-positive non-BRCA pathogenic variants and HRP, was significantly longer for niraparib (13.8 months: HR 0.62, 95% CI 0.50–0.76; 22.1 months: HR 0.40, 95% CI 0.27–0.62; 21.9 months: HR 0.43, 95% CI 0.31–0.59; 19.6 months: HR 0.50, 95% CI 0.31–0.83; and 8.1 months: HR 0.68, 95% CI 0.49–0.94, respectively) than for the control (8.2, 10.9, 10.4, 8.2 and 5.4 months, respectively) (3). Three reports made olaparib, olaparib plus bevacizumab and niraparib available for maintenance treatment in Japan. However, although these reports frequently compared each other, there have been no real-world reports that compared the three arms. Therefore, we conducted a retrospective study in a multicenter joint study.

In our study, the median PFS of patients who received maintenance treatment for platinum-sensitive advanced OC was 20.1 months. Patients with gBRCA pathogenic variants did not reach the median survival. Median PFS of patients with HRD was 20.2 months, 10.7 months for gBRCA wild-type and VUS, 7.2 months for HRP and 16.5 months for not analyzed patients. Patients with gBRCA pathogenic variants had a significantly better prognosis than the other groups. In the PAOLA-1 and PRIMA trials, BRCA pathogenic variants was also significantly prognostic for recurrence and death compared with non-specific clinical factors. (3,4). Similarly, in our study, both the univariate and multivariate analyses identified an association between gBRCA wild-type with poor prognosis.

Reports of AEs in the SOLO-1, PAOLA-1 and PRIMA trials showed high rates of hematological toxicities. In the SOLO-1 trial, anemia of grade 3 or higher occurred in 22% of patients, while neutropenia occurred in 8% and thrombocytopenia in 1% (2). In the PAOLA-1 trial, anemia of grade 3 or higher occurred in 17% of patients, while neutropenia occurred in 6% and thrombocytopenia in 2% (4). In the PRIMA trial, anemia of grade 3 or higher occurred in 31% of patients, while neutropenia occurred in 13% and thrombocytopenia in 29% (3). In our study, the rates of grade 3 or higher anemia, neutropenia and thrombocytopenia following maintenance treatment with olaparib, olaparib plus bevacizumab and niraparib were as follows: 33.3, 40.7 and 15.0% anemia, respectively; 16.7, 18.5 and 10.0% neutropenia, respectively; and 0.0, 7.4 and 6.7% thrombocytopenia, respectively. Regarding AEs resulting in drug interruption or discontinuation, olaparib- and olaparib plus bevacizumab-treated patients were significantly more anemic than those treated with niraparib, and niraparib-treated patients were significantly more thrombocytopenic than olaparib patients.

Renal function deterioration due to the use of PARP inhibitors has become a topic of discussion (8). In this study, we investigated hematological toxicity and renal function following chemotherapy and maintenance therapy among the three groups including bevacizumab. In olaparib plus bevacizumab-treated patients, anemia was significantly worse following maintenance treatment than that following chemotherapy. All three maintenance treatments worsened renal function significantly more than chemotherapy.

There are several limitations of this study, including its retrospective design. BRCA analysis was not performed in all HRD- and tBRCA-positive cases, and thus gBRCA evaluation could not accurately capture its frequency. Furthermore, renal function was only measured by GFR in this study, and additional renal function tests are recommended.

In conclusion, our study demonstrated that maintenance treatment was performed effectively and safely in platinum-sensitive advanced OC at multiple centers. Maintenance treatment in patients with platinum-sensitive advanced OC is likely to cause deterioration of renal function, and careful administration is essential.
